# An Integrated Analysis of Abattoir Lung Lesion Scores and Antimicrobial Use in Italian Heavy Pig Finishing Farms

**DOI:** 10.3390/ani14111621

**Published:** 2024-05-30

**Authors:** Matteo Recchia, Sergio Ghidini, Claudia Romeo, Federico Scali, Antonio Marco Maisano, Federica Guadagno, Silvio De Luca, Adriana Ianieri, Giovanni Loris Alborali

**Affiliations:** 1 Section Diagnostic and Animal Health, Istituto Zooprofilattico Sperimentale della Lombardia e dell’Emilia Romagna ‘Bruno Ubertini’ (IZSLER), Via Bianchi 7/9, 25124 Brescia, Italy; matteo.recchia@izsler.it (M.R.); federico.scali@izsler.it (F.S.); antoniomarco.maisano@izsler.it (A.M.M.); federica.guadagno@izsler.it (F.G.); giovanni.alborali@izsler.it (G.L.A.); 2 Department of Veterinary Medicine and Animal Sciences, University of Milan, Via dell’Università 6, 26900 Lodi, Italy; sergio.ghidini@unimi.it; 3 Center for Evolutionary Hologenomics—Globe Institute, University of Copenhagen, Øster Farimagsgade 5, 1353 Copenhagen, Denmark; 4 Independent Researcher, Chester CH1 2NH, UK; silvio.deluca@outlook.com; 5 Department of Food and Drug, Parma University, Via del Taglio 10, 43126 Parma, Italy; adriana.ianieri@unipr.it

**Keywords:** abattoir, AMU, lung scoring, pneumonia, porcine respiratory disease, stewardship program, surveillance

## Abstract

**Simple Summary:**

Abattoir lung lesion scoring is a common practice to assess the impact of respiratory diseases in pig production, but whether the information obtained could be used to optimize antimicrobial stewardship programs has rarely been investigated. In this study, lung and pleural scores collected at slaughter from Italian heavy pigs were compared with on-farm antimicrobial use during the six months prior to slaughtering. Lung scores were positively associated with the past use of antimicrobials considered critical for human medicine, suggesting that batches with worse scores may have been unsuccessfully treated with first-choice antimicrobials. This result emphasizes the role of abattoirs as strategic observatories for pig respiratory diseases, which may also be helpful for antimicrobial stewardship. The information obtained could provide useful feedback aiding the identification of gaps in biosecurity or inadequate vaccination plans, thus helping to reduce on-farm antimicrobial use.

**Abstract:**

Respiratory diseases significantly affect intensive pig finishing farms, causing production losses and increased antimicrobial use (AMU). Lesion scoring at slaughter has been recognized as a beneficial practice to evaluate herd management. The integrated analysis of abattoir lesion scores and AMU data could improve decision-making by providing feedback to veterinarians and farmers on the effectiveness of antimicrobial treatments, thus rationalizing their use. This study compared lung and pleural lesion scores collected at Italian pig slaughterhouses with on-farm AMU, estimated through a treatment index per 100 days (TI100). Overall, 24,752 pig carcasses, belonging to 236 batches from 113 finishing farms, were inspected. Bronchopneumonia and chronic pleuritis were detected in 55% and 48% of the examined pigs, respectively. Antimicrobials were administered in 97% of the farms during the six months prior to slaughter (median TI100 = 5.2), notwithstanding compliance with the mandatory withdrawal period. EMA category B (critical) antimicrobials were administered in 15.2% of cases (median TI100 = 0.06). The lung score was not associated with the total AMU, but significant, positive associations were found with the past use of critical antimicrobials (*p* = 0.041) and macrolides (*p* = 0.044). This result highlights the potential of abattoir lung lesion monitoring to rationalize antimicrobial stewardship efforts, contributing to AMU reduction.

## 1. Introduction

Slaughterhouses represent a crucial point in the meat supply chain as they play a pivotal role in identifying potential threats to animal and public health. The presence of permanent veterinary surveillance, as required by EU legislation (Regulation EU 2017/625; Regulation EU 2019/627), should guarantee continuous control aimed at identifying animals unfit for human consumption, which are consequently classified as by-products of animal origin [1]. Regardless of food safety and public health assurance, lesion scoring during post-mortem inspections at slaughter provides useful feedback to farmers and veterinarians on herd health and welfare conditions [2]. A recent Italian study on pigs’ health monitoring at slaughter showed that a large part of the variance in lesion scores is attributable to the farm effect, confirming the potential of abattoir lesion assessment to characterize farms’ health statuses [3]. These evaluations can also be used for epidemiological studies to investigate the temporal trends and geographical distributions of lesions, as well as to investigate risk factors for disease [4]. Compared to on-farm data collection, a health monitoring system at the abattoir level allows the analysis of a larger number of animals from different geographical areas, in a relatively short period of time and in a cost-effective way [1].

Several scoring systems for lesions in pig carcasses and organs have been developed over the last few decades [5]. The assessment of bites, scars or necrosis on the tail and the skin is commonly performed, as these have been described as ‘iceberg indicators’ of welfare issues on pig farms [6]. The evaluation of respiratory tract lesions is also of great interest, as the porcine respiratory disease complex (PRDC) represents a major economical, health and welfare problem in pig production worldwide [7]. Furthermore, PRDC is one of the main drivers of antimicrobial use (AMU) in pig farming [8]. The most prevalent lung lesions in slaughtered pigs are cranioventral pulmonary consolidation (CVPC) and chronic pleuritis, as reported in several studies [9,10,11]. CVPCs are typical findings in enzootic pneumonia (EP), a chronic condition primarily caused by *Mycoplasma hyopneumoniae* and affecting mainly grower–finisher pigs. EP-like lesions scored at slaughter have been positively associated with the herd *M. hyopneumoniae* seroprevalence at market weight [10]. However, these lesions are not pathognomonic for infections with *M. hyopneumoniae*, as they are also compatible with pulmonary infection by other respiratory pathogens, such as swine influenza virus and *Pasteurella multocida* [12]. The CVPCs observed at the slaughterhouse are often chronic, and the affected lung tissue can be retracted, forming scars or interlobular fissures [13]. Chronic pleuritis appears as fibrotic adhesions between the parietal and visceral membranes of the pleural sac and may result from the dissemination of pulmonary inflammation or be part of a polyserositis scenario. Such a condition is commonly detected at the abattoir, since the resolution of pleuritis can take three months or more, and, very often, this process is not completed prior to slaughter [14]. Dorso-caudal pleural lesions are highly suggestive of recovered pleuropneumonia primarily caused by *Actinobacillus pleuropneumoniae*, while cranio-ventral interlobar adhesions are strongly associated with complicated EP-like lesions [10].

Several standardized protocols have been developed to record and quantitatively assess bronchopneumonia at pig slaughter [13]. These are usually based on the visual inspection and palpation of the lungs and can be broadly classified into two-dimensional and three-dimensional methods. Two-dimensional scoring systems simply estimate the proportion of the lung surface area affected by the CVPC, whereas three-dimensional methods multiply this proportion by the relative weight of each lung lobe [15]. Due to the high working speed typical of industrial slaughterhouses, the choice of a scoring system that allows rapid assessment is preferred. The two-dimensional method proposed by Madec and Kobisch for the scoring of EP-like lesions is suitable for modern high-throughput pig slaughterhouses due to its simplicity and reliability [16], and its applicability has also been tested and confirmed in Italian heavy-weight pigs [17]. Similarly, the “Slaughterhouse Pleuritis Evaluation System” (SPES) proposed by Dottori et al. [18] is frequently applied in Italian pig abattoirs to evaluate chronic pleuritis. 

The information obtained is of paramount importance to detect subclinical lung infections and to evaluate the effectiveness of on-farm interventions to reduce the incidence of the respiratory disease, also considering the impact of the PRDC on AMU in pig production. Of note, national and international organizations have called for an urgent reduction in AMU in food-producing animals due to its contribution to the emergence and spread of antimicrobial resistance (AMR) [19,20,21]. To achieve this, a holistic approach comprising the improvement of biosecurity and the animal welfare status and the optimization of vaccination strategies and herd management is recommended [22,23,24,25,26]. However, there is still a general concern that a further reduction in AMU could jeopardize animal health and welfare due to the lack of or insufficient treatment of sick pigs, resulting in lower production outputs. The integrated analysis of AMU data and abattoir lesion scores could help to overcome such resistance, improving the decision-making by farmers and herd veterinarians [26,27].

With over 10 million pigs slaughtered per year [28], Italy has a well-established swine production system, mainly focused on rearing heavy pigs with a weight of 160–170 kg and at least nine months of age at slaughter [29]. Although AMU in Europe is decreasing, Italy is still one of the countries with the highest levels of antimicrobial consumption in livestock [30]. In 2015, high levels of AMU and the frequent use of antimicrobial classes considered critical for human medicine by various international organizations [31,32], such as quinolones and polymyxins, were described in Italian heavy pig finishing farms, albeit with large variations among farms [33]. High variability in AMU in Italian pig finishing farms was also reported by another study carried out from 2015 to 2017, which, moreover, found no significant decreasing trends [34]. 

Thus far, the relationship between lesions found at slaughter and AMU in pig pro-duction has rarely been investigated [8,27,35]. Further research in this field could shed light on whether and how information from abattoir lesion assessments can be used to improve antimicrobial stewardship, with the objective to reduce and rationalize AMU. Based on these considerations, the aim of the present study was to investigate and com-pare data on AMU with lung and pleural lesion scores, collected at slaughter, in Italian heavy pig finishing farms.

## 2. Materials and Methods

### 2.1. Evaluations at Slaughter 

Data collection was carried out from 2020 to 2022 in two high-throughput pig slaughterhouses located in Northern Italy. The two abattoirs shared a similar production flow and had a similar weekly output of about 15,000 heavy pigs intended for the production of Protected Designation of Origin (PDO) ham. Three trained veterinarians alternately performed assessments during this period using the same scoring methods. Bronchopneumonia lesions, suggestive of EP, were evaluated according to Madec’s grid [16,36]. Each lung lobe was scored from 0 to 4 based on the extent of the lesions, for a maximum individual score of 28. Pleuritis was scored using the SPES scoring, which classifies lesions according to their location, appearance and extent, for a maximum individual score of 4 [18]. Evaluations were conducted by visual inspection and palpation of the lungs at the official post-mortem inspection point, directly during the slaughtering process. Scores were registered using a voice recorder and subsequently reported in an Excel file. In order to reach a good level of agreement between assessors, all veterinarians were trained in the use of Madec’s grid and the SPES directly at the slaughter line. The training consisted of two full-weeks scoring sessions at both slaughterhouses (one week per abattoir) under the supervision of S.G. and G.L.A. Specific attention was paid to differentiation between lung lesions and the presence of artifacts, such as blood aspiration and scalding water lungs, which are reported as a frequent source of disagreement between observers [3,37]. The inter-rater reliability between observers was calculated using a subset of one hundred lungs collected during the two training sessions and evaluated simultaneously by the three veterinarians. The intraclass correlation coefficients (ICCs) for the EP-like lesion scores and SPES scores were both over 0.80 and deemed sufficient to start the data collection. In any case, during data analysis, evaluator IDs were always included in the statistical models to account for any inter-rater variability (see below). Details of the scoring systems used are given in Table 1.

A total of 236 batches from 113 intensive pig finishing farms, housing only finisher pigs intended for the production of PDO ham, were examined, for a total of 29,484 slaughtered heavy pigs. A batch (125 pigs on average) was defined as a group of animals from the same farm that were slaughtered on the same day and at the same abattoir. Ideally, a minimum of 100 pigs per batch were examined, resulting in 24,752 scored carcasses, with an average of 105 per batch. Batch-level EP and SPES scores (hereafter, proportional scores) were calculated as the sum of the individual scores within a batch on the number of examined carcasses multiplied by 28 and 4, as follows:

$$\frac{\sum_{i=1}^{n}{score}_{i}}{n\times maximum\;score}$$

Proportional scores could range from 0 to 1 and represented the proportion (or percentage) of the maximum theoretical score that a batch would reach if all individuals obtained the worst scores. Proportional scores were used in all of the following statistical analyses, but average standard batch scores (i.e., the sum of individual scores/the number of examined carcasses within a batch) are also presented to facilitate comparison with previous studies.

### 2.2. Estimation of Antimicrobial Use 

Since information was not available at the batch level, the AMU was estimated at the farm level considering the six months preceding the slaughter of each batch. This period corresponded to the growing–finishing phase of Italian heavy pigs, during which animals grow from 25–30 kg to a market weight of approximately 160–170 kg. All data required for the calculations were extracted from the Italian monitoring system ClassyFarm (www.classyfarm.it, accessed on 1 December 2023).

AMU was expressed as a treatment index 100 (TI100) considering the defined daily dose animal for Italy (DDDAit) as a metric, according to the standards described in a previous study on Italian heavy pig finishing farms [33]. The TI100 can be interpreted in three ways [38]: (1) as the percentage of time that an animal spends under treatment during its production cycle, (2) as the days spent under treatment for every 100 days of production, or (3) as the animals under treatment for every 100 animals present in a herd on any given day.

Antimicrobials belonging to EMA category B (‘restrict’) were considered critical—namely, third- and fourth-generation cephalosporins, polymyxins and quinolones [32].

### 2.3. Statistical Analysis

The relationships between the batch-level, proportional EP and SPES scores and on-farm AMU during the six months prior to slaughter were explored by means of mixed beta regressions. Preliminary Wilcoxon rank sum tests did not reveal any difference between the two sampled slaughterhouses in the EP (Z = 1.59; two-sided *p* = 0.11) or SPES scores (Z = 1.84; two-sided *p* = 0.07); hence, the slaughterhouse was not included in further analyses. For each of the two response variables, EP and SPES, two distinct models were run. In the first model, the total AMU was included as an explanatory variable. In the second model, the specific usage of critical antimicrobials and of the main antimicrobial classes used to treat respiratory infections (i.e., amphenicols, aminopenicillins, macrolides, pleuromutilins, sulfonamides and tetracyclines) was included. The farm size and year were included as covariates in all models, while the farm IDs and examiner IDs were included as random effects to account for repeated measures of the same farm and differences in lesion evaluations, respectively. All independent variables were standardized ([x-mean]/standard deviation) prior to analysis to improve the coefficient estimates. The significance level was set at *p* < 0.05. All analyses were carried out in the SAS 9.4 software (SAS Institute Inc., Cary, NC, USA).

## 3. Results

Bronchopneumonia lesions and chronic pleuritis were detected in approximately 55% and 48% of the examined pigs, respectively. The mean values of the proportional batch-level EP and SPES scores, batch size and farm size across all examined slaughterhouse batches (n = 236) are detailed in Table 2. Average standard scores are reported alongside the proportional score to facilitate comparisons with previous studies. The proportional EP and SPES scores were positively correlated (Spearman’s rho = 0.29; *p* < 0.0001).

**Figure 1:**
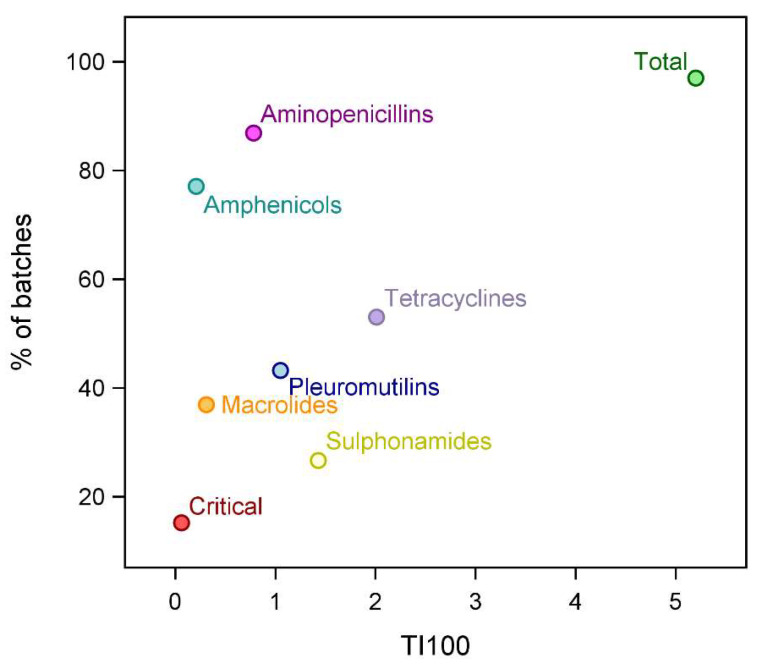
On-farm antimicrobial use (total and by class) of the pig batches examined for lung lesions at the slaughterhouse (n = 236): proportion of batches with use > 0 by median use values expressed as treatment index 100 (TI100). Antimicrobials were considered ‘critical’ when included in the European Medicines Agency’s category B. These were cephalosporins (third and fourth generation), polymyxins and quinolones [32].

During the six months before slaughtering, antimicrobials were administered in almost all farms (97.0%) of origin of the batches (median AMU = 5.2 TI100) (Table 3, Figure 1). Critical antimicrobials were administered in 15.2% of the farms, but in low quantities (median = 0.06 TI100). Among the classes administered to treat respiratory infections (Table 2), the two most frequently used were aminopenicillins and amphenicols, while sulfonamides and macrolides were less frequently administered, although the former were used sometimes in large quantities (up to 25.3 TI100).

**Figure 2:**
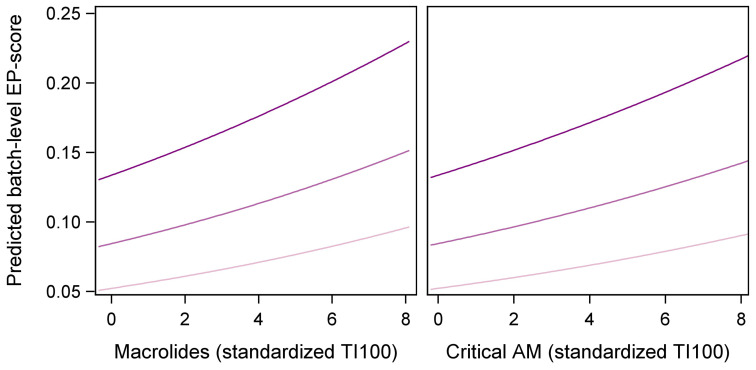
Predicted association between on-farm use of macrolides and critical antimicrobials (critical AM) on pigs’ enzootic pneumonia (EP) proportional scores at increasing (from light to dark purple) farm sizes. Antimicrobial use is expressed as standardized treatment index 100 (TI100).

The proportional EP score was not related to the total AMU and did not vary among the years, but it increased significantly, albeit slightly, with the increasing farm size (coefficient estimate ± ES: 0.16 ± 0.04; *p* < 0.0001). Among the antimicrobial classes, significant, positive associations of the EP scores with the past use of critical antimicrobials (0.07 ± 0.03; *p* = 0.041) and macrolides (0.08 ± 0.04; *p* = 0.044) were detected (Figure 2). The proportional SPES score was not related to any of the examined variables (all *p* > 0.05). 

## 4. Discussion

The present study aimed to analyze and compare abattoir lung and pleural lesion scores with on-farm antimicrobial usage during the six months prior to slaughter in Italian heavy pigs. Abattoir monitoring programs are a valuable tool to provide useful information to farmers and herd veterinarians that cannot be obtained from on-farm evaluations on living animals. Although lung scoring activities at slaughter are generally considered a relatively simple and rapid process, some important limitations may emerge, related to potential biases and feasibility. The selection of the most suitable scoring system is essential to collect high-quality data, and it must take into account the objectives of the assessment and its final use [39]. The reliability of the collected information depends on the reproducibility and objectivity of the selected method; therefore, scoring systems involving a certain degree of subjectivity should be avoided [40]. The implementation of commonly recognized and validated guidelines at a national and European level would allow for the collection of comparable and harmonized data across different countries, reducing the variability and ensuring consistency in the assessment process. To achieve this, a comprehensive training program for veterinarians is of paramount importance [39]. In addition, regular training and calibration sessions may be useful to ensure that assessors continue to score lung lesions properly over time. However, operator-dependent methods are difficult to implement continuously due to the increasing speed of slaughter lines and lack of human resources, hampering systematic lesion recording. In the near future, artificial intelligence (AI) technologies could adequately fulfill such a task, being more objective and repeatable in nature [41].

In this study, pig lungs were inspected for the presence of cranioventral pulmonary consolidation (CVPC) and pleuritis using Madec’s grid and the SPES, respectively. The prevalence of bronchopneumonia-affected lungs was around 55%, while chronic pleuritis was observed in approximately 48% of the examined pigs, both results being in line with previous studies [13]. The average standard EP score observed in our study (2.1) was similar to those reported by two previous Italian surveys [3,17], but was about twice as high as that reported in a third Italian study [10]. However, considering the seasonality of swine respiratory diseases, it should be noted that the latter only considered pigs slaughtered during warm months, when such infections are less common. Furthermore, the average standard SPES score (0.85) was comparable to those reported by the same authors [3,10]. These results suggest that, despite the efforts undertaken during the last few decades in implementing preventive measures and herd health management, respiratory diseases are still a major health problem in Italian pig finishing farming, influencing productivity and the use of antibiotics. As expected, the proportional EP and SPES scores were positively correlated. This correlation can be biologically justified by the etiopathogenesis of the two conditions. In fact, pleural inflammation can either originate as a primary event or be derived from severe, contiguous pulmonary inflammatory processes (secondary pleuritis). The etiological agents involved are generally able to trigger both types of lesions [42]. 

In almost all of the investigated farms (97%), antimicrobials were used during the six months prior to slaughter. It should be noted that this refers to the six months prior to slaughter outside the withdrawal period required by EU legislation for the delivery of animals to the slaughterhouse (Regulation EU 2019/6, Regulation EU 2010/37). The median AMU that we report is about half of that reported in a 2015 Italian study on the same type of farm [33]. This result seems to be in line with the general reduction in AMU in Italian animal production observed in the last few years [30]. Critical antimicrobials, namely third-and fourth-generation cephalosporins, polymyxins and quinolones (including fluoroquinolones) (category B antimicrobials [32]), were administered in a small percentage of farms (15.2%) and at relatively low usage values (0.06 TI100). The higher (16.7% of the total AMU) and more frequent (93.7% of the analyzed farms) use of the highest-priority critically important antimicrobials (HPCIAs) in Italian finishing pig farms was described during 2015 [33]. HPCIAs comprise third- and fourth-generation cephalosporins, polymyxins, quinolones and macrolides [31]. Although a direct comparison between studies is not possible since the WHO’s HPCIAs do not coincide with the EMA’s category B antimicrobials, it is nevertheless possible to state that the use of critical antimicrobials decreased in the three-year study period (2020–2022) compared to 2015.

As expected, aminopenicillins were the most used antimicrobial class to treat respiratory infections. Indeed, they are frequently prescribed in pig farms to treat respiratory infections caused by *Pasteurella multocida*, *Actinobacillus pleuropneumoniae*, *Trueperella pyogenes* and *Bordetella bronchiseptica* [43]. Furthermore, they are also commonly used to control swine systemic and enteric infections [44]. Amphenicols were the second most used class as they have broad-spectrum activity, being effective against aerobic and anaerobic Gram-positive and Gram-negative bacteria and *Mycoplasma* spp. Tetracyclines and macrolides were commonly administered too, as they are among the most frequently used antibiotics against *M. hyopneumoniae* infection [12]. However, classes such as lincosamides, pleuromutilins and aminoglycosides could represent an effective alternative to treat mycoplasmal pneumonia [12,45], particularly to reduce the use of macrolides. Although this class is not included in the EMA’s category B [32], macrolides are considered by the WHO to be among the highest-priority critically important antimicrobials for human medicine [31].

The relationship between AMU in pig finishing farms and lesions found at slaughter has rarely been investigated. Herd-level antimicrobial prescription data from Danish organic pig herds were analyzed for associations with abattoir meat inspection findings, but no significant results emerged [27]. In another study, no direct association was observed between the total AMU on Irish farrow-to-finish pig farms and the prevalence of pneumonia and pleurisy at slaughter, but positive associations were found with the prevalence of pericarditis, lung abscesses and liver milk spots [8]. In the present study, although the EP score did not vary with the total AMU, it was positively associated with the usage of macrolides and critical classes. The EMA’s category B antimicrobials should only be prescribed based on antimicrobial susceptibility testing results, when antibiotics belonging to categories C (e.g., macrolides, pleuromutilins, amphenicols) and D (e.g., aminopenicillins, tetracyclines, sulfonamides) are not clinically effective [32]. It is therefore possible that batches with worse EP scores, and thus characterized by the greater severity of respiratory diseases, were unsuccessfully treated with first-choice antimicrobials, consequently motivating the herd veterinarian to prescribe critical antimicrobials according to the cascade principle. Indeed, fluoroquinolones are indicated as a last-choice treatment for *M. hyopneumoniae*, as well as other respiratory infections [45].

From the opposite perspective, Pessoa et al. [35] explored the potential of incorporating on-farm animal-based welfare outcomes and AMU data within the Food Chain Information (FCI) to better predict organ lesions at slaughter, finding that the number of antimicrobial treatments may contribute to predicting both pneumonia and pleuritis. The FCI, which must accompany animals intended for slaughter in accordance with Regulation CE 2004/853, is a key tool for the implementation of an efficient risk-based meat safety assurance system, but its full potential is still not being realized due to shortcomings in its implementation [46,47]. It is essential to educate farmers and herd veterinarians on the importance of providing the slaughterhouse with more complete and significant information, which is needed to rationalize control actions and better protect consumer health. Furthermore, the exchange of information provided by the FCI is not unilateral. Indeed, Regulation CE 2004/853 requires FCI feedback from slaughterhouses to farms, which is essential to enable early and effective corrective action at the farm level. In this respect, lung scoring data could feed this information flow, and the inclusion of a color-coded farm risk level based on the lung scoring results could provide information that is easily interpreted by the farmer.

In addition to the AMU effects, the EP scores increased slightly but significantly with the farm size. The herd size is generally considered as a risk factor for respiratory diseases in pigs [48], although previous studies have shown conflicting results. Flesjå and Solberg found an increased prevalence of pneumonia in slaughtered pigs reared in larger herds [49], whereas, in another study, no significant associations could be detected between the herd size and the prevalence of mycoplasma-like lung lesions at slaughter [10]. The association observed in our study could be related to the greater difficulty in the early detection of clinical respiratory symptoms in larger groups of animals, resulting in delayed treatment and more severe lesions. This could be further aggravated by numerically inadequate farm personnel. However, it is important to consider that *M. hyopneumoniae* infections, and the resulting lung damage, are not always clinically detectable, and therefore clinical examination cannot be considered the only effective tool for the assessment of this pathology [17]. Another plausible reason for this association is the increased risk of transmission of *M. hyopneumoniae* in larger herds. The excessive density of pigs and the absence of an all-in, all-out (AIAO) herd management system can increase the circulation of this pathogen through close, usually nose-to-nose, contact between pen-mates. In the AIAO system, all of the same pigs are moved as a group through the different production stages. This avoids the mixing animals of different origins or ages, reducing stress and disease transmission [48]. 

Our study highlights lung lesion scoring at pig slaughter as a useful tool to gain preliminary insights into on-farm antimicrobial consumption. Although, of course, respiratory infections are not the only reason for antimicrobial treatment in finishers, they represent a major issue at this production stage. Hence, we believe that lung lesion scores may reflect most of the AMU during the months leading to slaughter with reasonable approximation. It must also be noted that our dataset lacked relevant information about farm-specific characteristics and management practices. Factors such as the animal density, biosecurity level, farm environment, vaccination protocols and clinical history may play a key role in characterizing the dynamics of antimicrobial use. Incorporating these variables into the analysis could significantly enhance the interpretation of the observed associations. In particular, future studies that include additional information on the herd status with respect to the major swine respiratory pathogens, such as *M. hyopneumoniae* and *A. pleuropneumoniae*, would allow a comparison between naïve and positive farms. Such a comprehensive approach could provide a more nuanced understanding of the complex interactions between antimicrobial consumption, animal health, and farm management, ultimately facilitating the development of evidence-based interventions to promote sustainable pig production and mitigate the risks associated with antimicrobial resistance.

## 5. Conclusions

This study was primarily aimed at understanding whether abattoir lung lesion scoring could provide any useful insights for the optimization of antimicrobial stewardship strategies. Although effective antimicrobial monitoring requires a more comprehensive approach, considering not only abattoir inspections but also on-farm assessments, our findings revealed that lung lesion scoring at slaughter could indeed be used as a preliminary tool to target interventions and resources by focusing efforts on the most critical farms, which are likely to have the worst management, health and welfare conditions. The rationalization of interventions is key to the success of a program aimed at reducing the overall use of antimicrobials, and lesion scoring at slaughter appears to be a relatively simple tool for risk-based farm assessment. Of course, the reliability of the information obtained at the abattoir is of paramount importance and scoring systems that involve some degree of subjectivity should be avoided. A comprehensive training program using standardized definitions of lesions is recommended, as well as regular calibration sessions to ensure the reliability of the data over time. New technologies, such as artificial intelligence-based methods, may provide a unique tool to systematically analyze the respiratory health statuses of slaughtered pigs, thus allowing for the collection of more reliable and standardized data.

## Figures and Tables

**Table 1:** Pleuritis and enzootic pneumonia (EP)-like lesion scoring systems.

Lesion	Description	Score	
EP-like lesions	Red to purplish areas of lung consolidation with increased firmness, usually with a cranioventral pattern	0	No lesions
		1	Lesion affecting < 25% of the lobe surface
		2	Lesion affecting 25–49% of the lobe surface
		3	Lesion affecting 50–74% of the lobe surface
		4	Lesion affecting ≥ 75% of the lobe surface
Pleuritis	Fibrinous or fibrous (chronic) pleural lesion	0	No lesions
		1	Adhesions between cranio-ventral portions of lung lobes or monolateral mild adhesions at the ventral margin of a diaphragmatic lobe
		2	Dorsocaudal unilateral focal pleuritis
		3	Bilateral pleuritis of type 2 or extended unilateral pleuritis
		4	Severely extended bilateral pleuritis

**Table 2:** Mean values and their 95% confidence intervals for proportional and standard batch-level scores describing enzootic pneumonia-like lesions (EP score) and pleuritis (SPES score) across all pig batches (n = 236) examined at the slaughterhouse. The median batch and farm size (expressed as the number of pigs) and their interquartile range (IQR) are also reported.

Variable	Proportional Score		Standard Score		Median	IQR
	Mean	95%CI	Mean	95%CI		
Batch-level EP score	0.07	0.07–0.08	2.07	1.91–2.24		
Batch-level SPES score	0.21	0.20–0.22	0.85	0.80–0.90		
Batch size	-	-	-	-	130	15
Farm size	-	-	-	-	3041	5176

**Table 3:** Total antimicrobial use (AMU), use of critical antimicrobials and use of main antimicrobial classes against respiratory infections in the examined pig batches (n = 236); values expressed as treatment index 100 (TI100). Median values, interquartile ranges and minimum and maximum values refer only to batches where on-farm AMU > 0.

AMU	% of Batches with On-Farm AMU > 0 (n)	TI100			
		Median	Interquartile Range	Minimum	Maximum
Total	97.0% (229)	5.25	7.06	0.017	45.33
Critical classes *	15.2% (36)	0.06	0.51	0.003	2.60
Amphenicols	77.1% (182)	0.21	0.43	0.002	5.34
Aminopenicillins	86.9% (205)	0.78	2.14	0.002	19.03
Macrolides	36.9% (87)	0.31	0.57	0.013	5.99
Pleuromutilins	43.2% (102)	1.04	2.49	0.001	12.76
Sulfonamides	26.7% (63)	1.43	2.71	0.001	25.31
Tetracyclines	53.0% (125)	2.10	3.17	0.014	16.49

* European Medicines Agency category B antimicrobials: cephalosporins (third and fourth generation), polymyxins, quinolones [32].

## Data Availability

The data included in this study can be provided by the corresponding author upon reasonable request.
